# Correlation between gut microbiota composition, enteric infections and linear growth impairment: a case–control study in childhood stunting in Pidie, Aceh, Indonesia

**DOI:** 10.1186/s13099-023-00581-w

**Published:** 2023-11-09

**Authors:** Tristia Rinanda, Catur Riani, Anita Artarini, Lucy Sasongko

**Affiliations:** 1 Department of Pharmaceutics, School of Pharmacy, Institut Teknologi Bandung, Ganesha 10, Bandung, 40132 West Java Indonesia; 2 Department of Microbiology, Faculty of Medicine, Universitas Syiah Kuala, Darussalam, Banda Aceh, 23111 Aceh Indonesia

**Keywords:** Stunting, Gut microbiome, Dysbiosis, IGF-1, Enteric infection

## Abstract

**Background:**

Gut microbiota is pivotal in maintaining children's health and well-being. The ingestion of enteric pathogens and dysbiosis lead to Environmental Enteric Dysfunction (EED), which is essential in stunting pathogenesis. The roles of gut microbiome and enteric infections have not been explored comprehensively in relation to childhood stunting in Indonesia. This study aimed to determine the correlation between gut microbiota composition, enteric infections, and growth biomarker, Insulin-like Growth Factor 1 (IGF-1), in stunted children from Pidie, Aceh, Indonesia.

**Methods:**

This study was a case–control study involving 42 subjects aged 24 to 59 months, comprising 21 stunted children for the case and 21 normal children for the control group. The IGF-1 serum level was quantified using ELISA. The gut microbiome profiling was conducted using 16S rDNA amplicon sequencing. The expression of enteric pathogens virulence genes was determined using quantitative PCR (qPCR) assay. The correlations of observed variables were analysed using suitable statistical analyses.

**Results:**

The result showed that the IGF-1 sera levels in stunted were lower than those in normal children (p ≤ 0.001). The abundance of Firmicutes (50%) was higher than Bacteroidetes (34%) in stunted children. The gut microbiome profile of stunted children showed enriched genera such as *Blautia, Dorea, Collinsella, Streptococcus, Clostridium* sensu stricto 13*, Asteroleplasma* and *Anaerostipes.* Meanwhile the depleted genera comprised *Prevotella, Lactococcus, Butyrivibrio, Muribaculaceae, Alloprevotella, Akkermansia*, *Enterococcus, Terrisporobacter* and *Turicibacter.* The abundance of water biological contaminants such as *Aeromonas, Stappiaceae, *and *Synechococcus* was also higher in stunted children compared to normal children. The virulence genes expression of Enteroaggregative *Escherichia coli* (*aaiC*), Enterotoxigenic *E. coli* (*estA*), Enteropathogenic *E. coli* (*eaeA*), *Shigella*/Enteroinvasive *E. coli* (*ipaH3*) and *Salmonella enterica* (*ompC*) in stunted was higher than in normal children (p ≤ 0.001), which negatively correlated to height and level of IGF-1.

**Conclusion:**

The present study showed the distinctive gut microbiome profile of stunted and normal children from Pidie, Aceh, Indonesia. The gut microbiota of stunted children revealed dysbiosis, comprised several pro-inflammatory, metabolic abnormalities and high-fat/low-fiber diet-related taxa, and expressed virulence genes of enteric pathogens. These findings provide evidence that it is imperative to restore dysbiosis and preserve the balance of gut microbiota to support linear growth in children.

**Supplementary Information:**

The online version contains supplementary material available at 10.1186/s13099-023-00581-w.

## Background

Stunting or linear growth faltering is the most prevalent form of undernutrition. To date, 149.2 million children under five years are affected by stunting worldwide, and more than half of those children live in Asia. The Covid-19 pandemic may substantially increase the number due to limitations in accessing adequate nutrition and healthcare services [1]. Indonesia is one of the Asian countries still struggling to eradicate stunting. In 2022, Indonesia was fourth on the list of the countries with the highest prevalence of stunting, reaching 21.6% [2].

Stunting is more than just growth impairment. This undernourished state also deteriorates intellectual and cognitive ability. The quality of health will also decline due to future metabolic complications such as diabetes and other cardiovascular diseases. These will affect the quality of human resources and become significant threats to the nation's future. The devastating impacts of stunting can last for a lifetime and affect the next generation [1].

Previous studies have revealed the role of gut microbiota in the pathogenesis of stunting. The gut microbiota imbalance, namely dysbiosis, was the underlying mechanism caused by the chronic ingestion of enteric pathogens. The existence of enteric pathogens activated the gut immune system and led to local and systemic inflammation. These conditions disturb gut physiology by reducing the function of the gut barrier, increasing permeability, and impairing the gut structure. These states were defined as Environmental Enteric Dysfunction (EED). EED causes the impairment of nutrient absorption, resulting in nutrient deficiency [3].

A worldwide study revealed that childhood diarrhoea was one of the major causes of stunting in developing countries [4]. World Health Organization has proposed enteric infections and diarrhoeal diseases as contributing factors to stunting. Most studies in Indonesia revealed a strong correlation between stunting in children under 5 years of age with diarrhoeal episodes [5]. Previous studies revealed the common enteric pathogens associated with diarrhoea and linear growth impairment in children, such as Enteroaggregative *Escherichia coli* /EAEC [6], Enterotoxigenic *E. coli*/ETEC [7, 8], Enteropathogenic *E. coli*/EPEC [9], Enteroinvasive *E. coli*/EIEC [10], *Shigella* [6], and *Salmonella* [11].

Insulin-like Growth Factor 1 (IGF-1) is the most common growth biomarker used in studying linear growth impairment. The previous studies revealed the correlation between the low concentration of IGF-1, the increase of inflammation markers, and the presence of enteric infections in stunted children [12–16]. The secretion of IGF-1 was also a result of gut microbiota modulation activity, which was confirmed by previous animal studies [17–19]. The role of IGF-1 in linear growth in humans was indicated by the activity of IGF-1in supporting bone growth through the Growth Hormone (GH)-IGF-1 axis [20–22]. The low level of IGF-1 was also associated with several non-communicable diseases, such as diabetes [23]. Therefore, IGF-1 could be developed as a potential predictor or marker for long-term health status in children [24].

Stunting is widely distributed in Indonesia and Aceh is one of the provinces with a high prevalence of stunting, which is 33.2% in 2021 [2]. Not only does it have to deal with stunting, but Aceh also struggles with other health-related issues. A high prevalence of diarrhoea among toddlers was also reported in Aceh, reaching 13.8% in 2019. This number was higher than the national prevalence, which was 11%. Aceh was also the province with the lowest basic immunization coverage in Indonesia, reaching 50.9% [25]. The location of this study, Pidie district, was one of the areas in Aceh with high stunting cases [2]. It has been designated as one of the stunting loci in Indonesia [26].

The vast implication of the stunting impacts on health and development of this nation demands some progressive measures. Several established stunting eradication programs have successfully lowered the prevalence of stunting. The nutritional approaches have been mainly focused on the supplementation of macronutrients such as carbohydrates and protein and micronutrients such as zinc, vitamin A, and iron [27]. The success of supplementation relies significantly on the gut absorption function, which declines due to dysbiosis and EED in the case of stunting.

This study aimed to determine the correlation between gut microbiota composition, enteric infections, and growth biomarker, Insulin-like Growth Factor 1 (IGF-1), in stunted children from Pidie, Aceh, Indonesia. To our knowledge, the microbiome study of stunted children in Aceh has never been published. The results of this study are essential in capturing certain types of gut microbiota that play a pivotal role in linear growth. It provides beneficial information since gut microbiota exhibits dynamic features related to gender, age, diet, geographical regions, socio-demographic conditions, and health status. These findings may support the more comprehensive approaches to stunting prevention and eradication.

## Results

### Characteristics of subjects

A total of 42 subjects consisting of 21 stunted and 21 normal children aged 24 to 59 months were enrolled in this study (Table 1). All subjects in the case group were categorized as stunted based on WHO growth standards [28]. The birthweight and BMI between observed groups was not statistically different. Most stunted children experienced bloody or watery diarrhoea (86%) and had incomplete basic immunization status (71%). The IGF-1 sera level in stunted children was lower than in normal children (Table 1).

**Table 1:** Characteristics and IGF-1 concentration of observed groups

Parameter	Normal (n = 21)	Stunted (n = 21)	*p*-value
Gender, female/male	11/10	11/10	
Age, mean ± SD (months)	39.9 ± 10.3	40.14 ± 10.4	
Height, mean ± SD (cm)	93.6 ± 6	88.2 ± 5.3	*0.004
Weight, mean ± SD (kg)	13.4 ± 1.8	12.3 ± 1.5	*0.048
Birthweight, mean ± SD (kg)	3.3 ± 0.5	3.0 ± 0.5	0.052
BMI, mean ± SD (kg/m.^2^)	15.9 ± 1.3	15.2 ± 1.1	0.121
IGF-1, median, IQR (ng/ml)^#^	95.9 (139.8)	18.87 (48.6)	*0.001
History of diarrhoea ^			* < 0.001
Yes	4 (19%)	18 (86%)	
No	17 (81%)	3 (14%)	
Basic immunization status^			* < 0.001
Complete	19 (90%)	6 (29%)	
Incomplete	2 (10%)	15 (71%)	

All T-test except ^#^Mann–Whitney and ^Fisher-Exact Test

*p < 0.05

### Gut microbiome composition in stunted and normal children

**Fig. 1:**
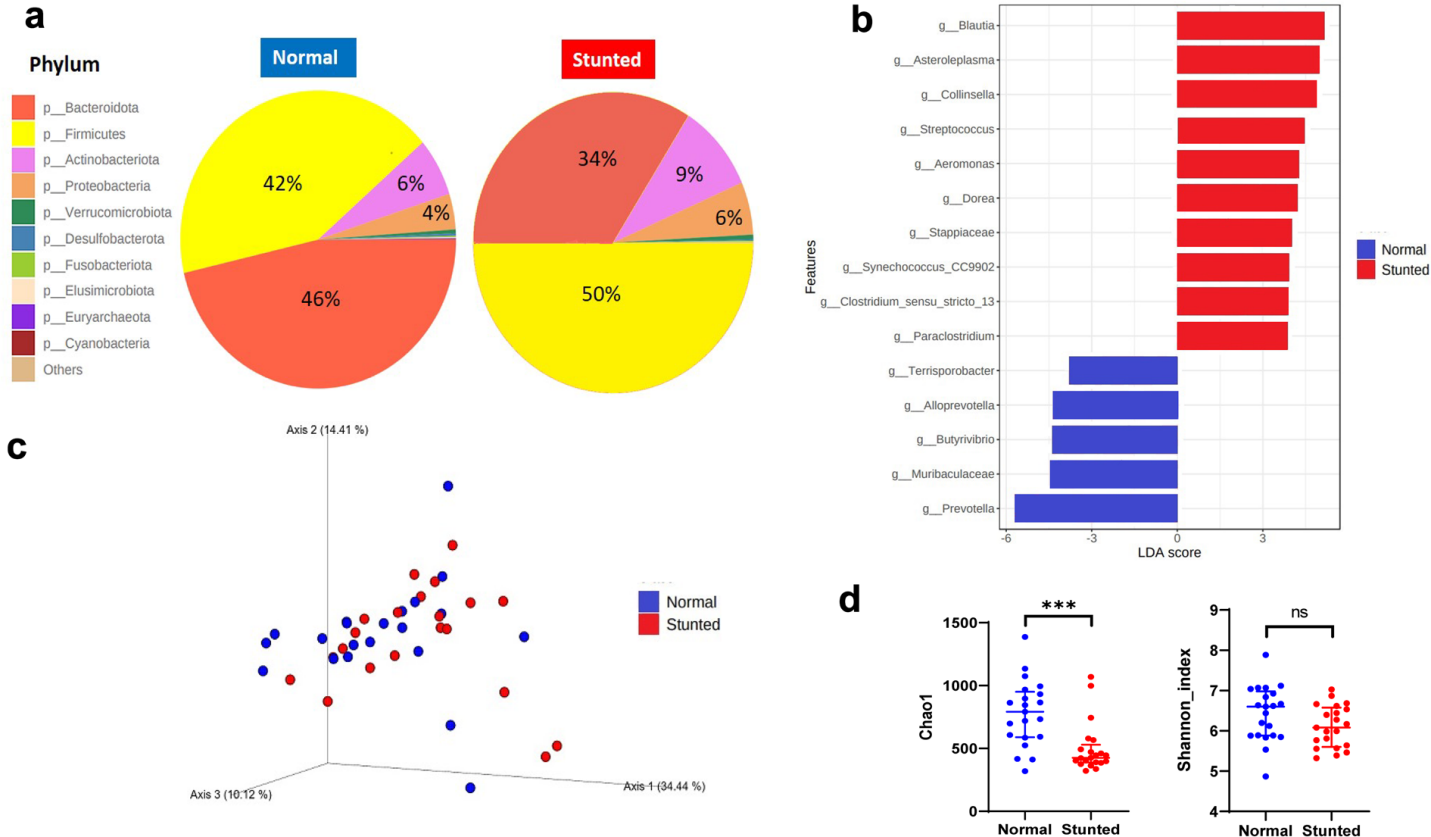
Gut microbiota composition in normal and stunted children. **a** Relative abundance of bacterial phyla. **b** Significant different genera in stunted and normal children (LDA score > 3) **c** Weighted UniFrac model of beta diversity. **d** Chao1 and Shannon index of alpha diversity. The statistically significant difference was indicated by an asterisk (ns not significant; ***p ≤ 0.001)

The human gut microbiome comprises a complex microbial community that changes temporally and spatially, and in relation to health status [29]. The 16S rDNA amplicon sequencing is the most extensively used approach in determining gut microbiota composition [30]. A total of 14,423 Amplicon Sequence Variant (ASV) were observed in 16S rDNA amplicon sequencing and proceeded to bioinformatics analyses. Phylum-level abundance analysis showed a predominant portion of Firmicutes compared to the Bacteroidetes in stunted children (Fig. 1a), resulting in an increasing Firmicutes to Bacteroidetes (F/B) ratio. Differential abundance analysis of gut microbiome (Linear Discriminant Analysis (LDA) score > 3) in stunted children revealed the significant abundance of *Stre*p*tococcus, Blautia, Dorea, Collinsella*, *Clostridium *sensu stricto 13, *Aeromonas, Stappiaceae,* and *Synechococcus* CC9902 (Fig. 1b). Meanwhile, the important taxa in normal children comprised of *Prevotella, Butyrivibrio, Muribaculaceae*, and *Alloprevotella* (Fig. 1b). Weighted UniFrac (Fig. 1c) and Unweighted UniFrac (Additional file 1: Fig. S1) models of beta diversity showed the different structures of the gut microbiome community among observed groups (p = 0.042 and p = 0.001). Chao1 and Shannon index represented the gut microbiome diversity among samples/individuals. The rarefaction curve showed sufficient sequencing depth in this study (Additional file 1: Fig. S2). Chao1 value indicated the species richness, which was lower in stunted children than in normal children. Shannon index showed more complex species diversity, and it was not statistically different between observed groups (Fig. 1d).

Spearman analysis revealed the depleted and enriched taxa in stunted children (Table 2). Several depleted taxa were *Prevotella, Lactococcus, Butyrivibrio, Muribaculaceae, Alloprevotella, Akkermansia*, *Enterococcus, Terrisporobacter* and *Turicibacter.* In the other hand, *Blautia, Dorea, Collinsella, Asteroleplasma, Streptococcus, Lactobacillus, Anaerostipes, Aeromonas, Stappiaceae,* and *Synechococcus* CC9902 were found to be enriched in stunted children.

**Table 2:** Genus-level differences between stunted and normal children

Depleted in stunted children			Enriched in stunted children		
Genus	p-value	Adjusted p-value	Genus	p-value	Adjusted p-value
*Prevotella*	0.037	0.090	*Blautia*	< 0.001	0.004
*Lactococcus*	< 0.001	< 0.001	*Collinsella*	0.015	0.059
*Butyrivibrio*	< 0.001	0.003	*Dorea*	0.024	0.073
*Muribaculaceae*	< 0.001	0.001	*Asteroleplasma*	0.007	0.031
*Alloprevotella*	0.004	0.019	*Lactobacillus*	0.004	0.019
*Akkermansia*	0.006	0.029	*Streptococcus*	0.004	0.019
*Enterococcus*	0.036	0.090	*Anaerostipes*	0.042	0.091
*Terrisporobacter*	0.025	0.075	*Synechococcus* CC9902	< 0.001	< 0.001
*Turicibacter*	< 0.001	0.001	*Aeromonas*	0.001	0.006
*Clostridia* UCG-014	0.003	0.018	*Stappiaceae*	< 0.001	< 0.001
*Methanobrevibacter*	0.025	0.075	*Marvinbryantia*	0.028	0.082
*Elusimicrobium*	0.015	0.060	Ruminococcaceae UBA1819	0.026	0.079
*Fournierella*	< 0.001	0.004	*Paraclostridium*	< 0.001	0.001
Ruminococcaceae CAG-352	0.001	0.005	*Nautella*	< 0.001	< 0.001
*Anaerovibrio*	0.002	0.013	*Clostridium *sensu stricto 13	< 0.001	< 0.001
*Faecalibaculum*	0.005	0.022	*Clostridium *sensu stricto 13	< 0.001	< 0.001
Prevotellaceae NK3B31 group	0.002	0.013	Halieaceae OM60(NOR5) clade	< 0.001	0.001
Coriobacteriaceae UCG-002	0.010	0.040	Uncultured Chitinophagales	< 0.001	0.001
Unassigned Enterobacteriaceae	0.016	0.061	Unassigned Cyanobiaceae	< 0.001	0.004
Uncultured Oscillospiraceae	0.018	0.063	*Candidatus actinomarina*	< 0.001	0.002
*Parasutterella*	0.016	0.060	*Shewanella*	< 0.001	0.001
*Dubosiella*	0.027	0.081	Actinobacteria PeM15	< 0.001	< 0.001
*Mucispirillum*	0.040	0.090	*Erythrobacter*	< 0.001	< 0.001
*Eubacterium nodatum group*	0.020	0.063	*Candidatus aquiluna*	< 0.001	< 0.001
Butyricicoccaceae UCG-009	0.008	0.034	Uncultured Saprospiraceae	< 0.001	< 0.001
Ruminococcaceae	0.040	0.090	*Morganella*	< 0.001	< 0.001
*Ochrobactrum*	0.020	0.063	Unassigned Rhodobacteraceae	< 0.001	0.001
*Granulicatella*	0.033	0.090	*Flavonifractor*	< 0.001	0.002
Lachnospiraceae UCG-006	0.040	0.090	*Eubacterium xylanophilum* group	0.002	0.013
*Rikenella*	0.040	0.090	Flavobacteriaceae NS4 marine group	0.002	0.013
*Solobacterium*	0.040	0.090	*Thalassococcus*	< 0.001	0.002
*Ruminobacter*	0.040	0.090	*Peptostreptococcus*	< 0.001	0.002
Unassigned Anaerovoracaceae	0.040	0.090	*Haemophilus*	0.002	0.013
*Butyricimonas*	0.040	0.090	Flavobacteriaceae	0.000	0.001
Uncultured Christensenellaceae	0.040	0.090	*Methylophaga*	0.005	0.022
*Cutibacterium*	0.005	0.002	Unassigned Microbacteriaceae	< 0.001	0.004
			*Sporacetigenium*	< 0.001	0.002
			*Cognatishimia*	0.002	0.013
			Rhodobacteraceae HIMB11	0.001	0.007
			*Alteromonas*	0.003	0.018
			*Labrenzia*	0.001	0.007
			*Eubacterium*	0.006	0.029
			*Lysinibacillus*	0.001	0.007
			Unassigned Clostridiaceae	0.001	0.007
			*Halomonas*	0.005	0.022
			*Plesiomonas*	0.020	0.013
			*Coxiella*	< 0.001	0.001
			*Vibrio*	0.002	0.013
			Alphaproteobacteria SAR11 Clade III	0.001	0.007
			Gammaproteobacteria EV818SWSAP88	0.010	0.04
			*Lewinella*	0.010	0.04
			Uncultured Cryomorphaceae	0.005	0.022
			Sphingobacteriales NS11-12 marine group	0.002	0.013
			*Proteus*	0.000	0.004
			Unassigned Flavobacteriaceae	0.002	0.013
			C*lostridium *sensu stricto 9	0.020	0.063
			*Bacillus*	0.018	0.063
			*Luminiphilus*	0.016	0.060
			Uncultured Flavobacteriaceae	0.030	0.088
			*Fulvimarina*	0.020	0.063
			*Rhodopirellula*	0.020	0.063
			*Intestinimonas*	0.040	0.090
			*Balneola*	0.020	0.063
			*Marinobacter*	0.020	0.063
			*Oceanicaulis*	0.020	0.063
			*Streptococcus*	0.004	0.019
			Vampirovibrionales	0.020	0.063

p-value < 0.05

Adjusted p-value < 0.1

### Difference expression of virulence genes among observed groups

**Fig. 2:**
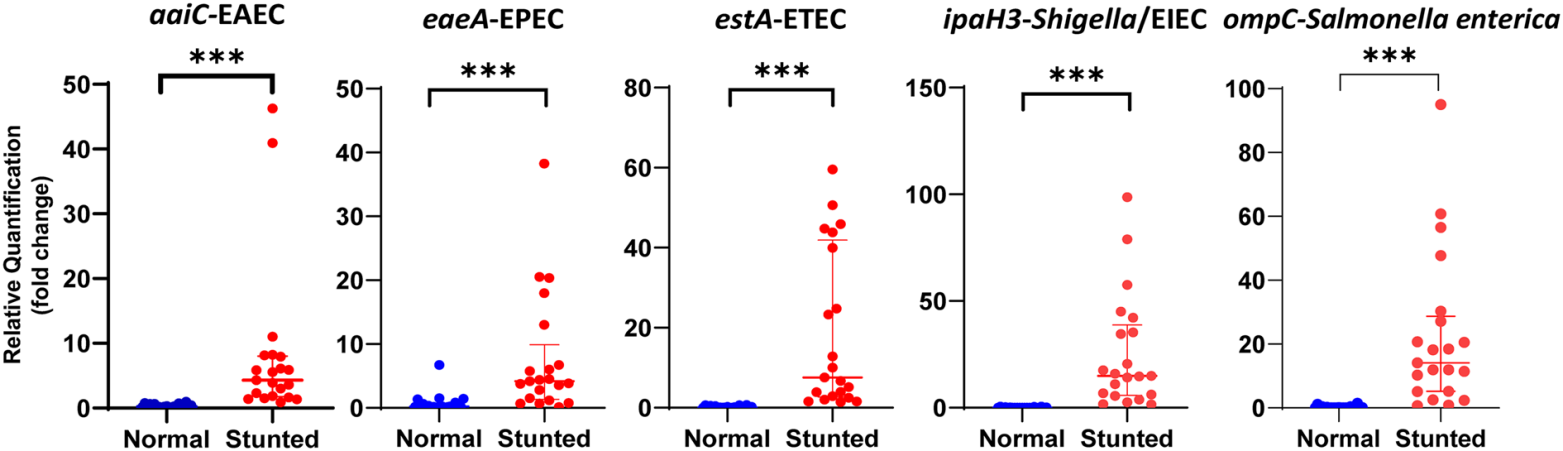
Virulence genes expression in stunted and normal children. Statistical analysis was performed using Mann–Whitney U Test (p < 0.05). The statistically significant difference was indicated by an asterisk (***p ≤ 0.001)

The result of 16S rDNA amplicon sequencing revealed the presence of several taxa related to enteric infections, such as *Escherichia-Shigella* and *Salmonella* (Additional file 2). Pathogenic bacteria express virulence factors to invade the host cells and retain intracellular survival, persistence, and spreading [31]. The present study used qPCR assay to determine the expression level of virulence genes from several enteric pathogens by measuring the relative quantification. The measurement revealed the higher expression of virulence genes of enteric pathogens in stunted children compared to normal children as follows: *aaiC* expression of EAEC (p < 0.001); *eaeA* expression of EPEC (p < 0.001); *estA* expression of ETEC (p < 0.001); *ipaH3* expression of *Shigella*/EIEC (p < 0.001); and *ompC* expression of *Salmonella enterica* (p < 0.001)(Fig. 2).

### Correlation between virulence gene expression and linear growth

**Fig. 3:**
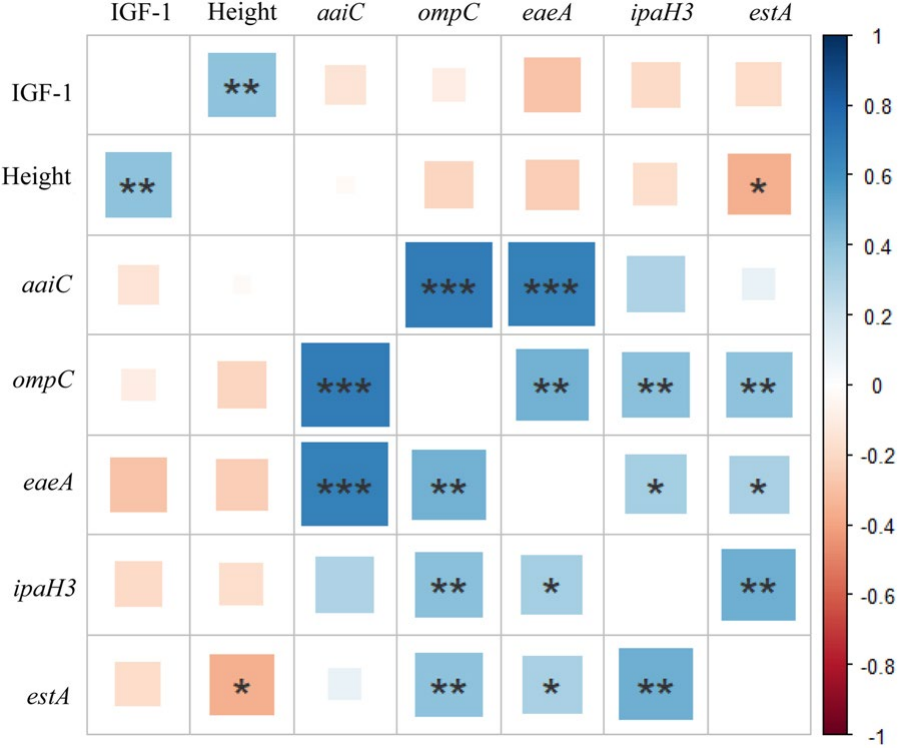
Correlation between the expression of virulence genes and level of IGF-1. The blue colour indicated a positive correlation, and the red showed a negative correlation. The analysis was performed using Spearman-rank test and statistically significant correlation was marked by an asterisk (*p ≤ 0.05; **p ≤ 0.01; ***p ≤ 0.001)

The ingestion of enteric pathogens lead to dysbiosis and linear growth impairment [32]. The correlation matrix was used to visualize the correlation between the virulence genes expression, IGF-1, and height (Fig. 3). IGF-1 level was significantly associated with height (p = 0.007). There was a negative correlation between virulence genes expression and IGF-1 sera level, although it was not statistically significant. The expression of *estA*, gene encoding ETEC heat-stable enterotoxin, was negatively correlated to height (p = 0.020). There was a positive correlation among each virulence gene of enteric pathogen observed in this study as follows: the *aaiC* expression was positively correlated to *ompC* and *eaeA* expression (p < 0.001); the *ompC* expression was positively correlated to *eaeA* (p = 0.001), *ipaH3* (p = 0.008) and *estA* expression (p = 0.006); the *eaeA* expression was positively correlated to *ipaH3* (p = 0.03), and *estA* expression (p = 0.03); and the *ipaH3* expression was positively correlated to *estA* (p = 0.001) (Fig. 3).

## Discussion

In accordance with the previous reports, this study showed a significant correlation between the history of diarrhoea and stunting [33–35]. Diarrhoea could be one of the clinical manifestations of EED, representing enteric pathogens exposure, although the exposure could be present with or without diarrhoea. Diarrhoea causes electrolyte imbalance and water loss, leading possibly to nutrition depletion [3]. High energy expenditure, malabsorption, and lower appetite due to diarrhoea contribute to malnutrition [36]. A dissimilar result from another study in Indonesia showed no association between a history of diarrhoea and stunting since it was only an acute episode; therefore, there was no significant effect on linear growth [37]. Subclinical infections have raised a concern about their contribution to linear growth retardation. The absence of recognized episodes of diarrhoea (three or more formless stools per day) and asymptomatic carriages of known enteric pathogens might lead to subclinical gut inflammation and impaired linear growth [38]. A study showed that atypical EPEC could retain gut colonization due to the absence of the *bfpA* gene. The infections commonly emerge as mild and subclinical, although some cases report more severe clinical symptoms [39].

Basic immunization in early life is fundamental for children to reach optimal growth. A secondary analysis study from 13 provinces in Indonesia demonstrated a positive correlation between incomplete immunization status and stunting in children aged 2 to 5 years [40]. A study in Thai children also revealed a similar outcome [41]. An extensive study in Africa showed that early-life vaccination decreased the odds of stunting and anemia. The unvaccinated children might be prone to recurring infections leading to prolonged inflammation [42]. A longitudinal study in Zimbabwe revealed that the prolonged inflammation in stunted infants was negatively correlated to IGF-1 levels and erythropoietin [12].

This study also revealed lower levels of IGF-1 sera in stunted children than in normal children. A study of stunted children aged 4 to 10 in Egypt also revealed a declined level of IGF-1 compared to the controls [15]. Another study in Indonesia showed a positive association between low IGF-1 levels and stunting in children with transfusion-dependent thalassemia [16]. The pro-inflammatory markers in stunting reduced the circulating IGF-1, impairing linear growth [12–14]. Children with higher levels of IGF-1 during the first year of life were less likely to develop stunting [14]. However, a different result showed that IGF-1 levels were independently associated with stunting in Bangladeshi children aged 12 to 18 months [43].

The present study used IGF-1 as a growth biomarker in stunting since it was more sensitive to nutritional deficiency than another biomarker, namely IGF Binding Protein 3 (IGFBP3) [44]. The IGF-1 was more stable than GH and also unaffected by diurnal fluctuations and pre-analytical factors such as food intake, exercise, and stress proximately before blood sampling [45]. Free form of IGF-1 in serum, as was used in this study, demonstrated more prominent physiological activity and clinical relevance than total IGF-1 (bound and free form) [46]. A concern has been raised due to the low concentration of free IGF-1 serum in several catabolic conditions such as malnutrition, anorexia nervosa, and poorly controlled type 1 diabetes, as well as in hypothyroidism [45]. The concern was no longer applied since these conditions were not found in all subjects recruited. Further research is needed to elaborate on the relationship between IGF-1 levels and stunting.

The increased F/B ratio in this study was also found in other reports, such as in West Java [47] and Chimalhuacán, Mexico. The higher abundance of Firmicutes than Bacteroidetes indicated dysbiosis and inflammation, and it was strongly correlated to high sugar-low fiber intake and lipid metabolism impairment, resulting in childhood obesity [48].

The high abundance of several taxa in stunted children also indicated dysbiosis, inflammation, and an imbalanced diet. *Blautia* was associated with high levels of long-chain triglycerides and a pre-diabetic state in patients with Type I Diabetes Mellitus. *Blautia* and *Dorea* represented a high fat intake and significantly correlated to inflammation and the impairment of the gut barrier [49]. *Blautia* and *Clostridium *sensu stricto 13 were positively correlated to serum lipids [50]. *Streptococcus* was a pro-inflammatory microbiota and commonly found in systemic inflammation states. *Collinsella* was associated with low-fiber intake, hyperglycemia state, and decreased intestinal integrity [51]. The presence of metabolic abnormalities-related taxa may become a predictor of future metabolic complications in stunted children.

Interestingly, this study found a higher abundance of water biological contaminants in stunted children than in normal children, such as *Aeromonas*, *Stappiaceae,* and *Synechococcus* CC9902. *Aeromonas* is commonly known as fish pathogens and naturally reside in aquatic environments. This organism has gained a warning on human health due to its ability to colonize and infect humans. Acute gastroenteritis was a common clinical manifestation of *Aeromonas* infection [52]. A study revealed *Aeromonas* as the significant pathogens in moderate to severe diarrhoea in children from Bangladesh and Pakistan, and it was also associated to the degree of stunting in children aged 24 to 59 months. *Aeromonas*-associated diarrhoea was co-occurred with dysentery caused by *Shigella* spp. [53]. *Stappiaceae* is an alphaproteobacterium that resides in marine environments, especially in mangrove sediments. It is commonly found in shrimps and oysters [54]. *Synechococcus*, one of the cyanobacteria, has been known as a seafood contaminant. The contamination occurred due to the accumulation of cyanotoxins on the seaweed, crustaceans, or fish. A high accumulation of toxins could harm humans [55]. The cyanotoxin-producing *Synechococcus* spp. has been reported on freshwater environments in Singapore. This organism produced cylindrospermopsin (CYN) and anatoxin-a [56]. The CYN of Cyanobacteria showed effects on liver, small intestine and colon. The lipopolysaccharides of Cyanobacteria affected the intestinal and immune cells, although the impacts on humans were still debatable [57]. Since the study site was located near the coastal area and most subjects consumed marine products as a primary source of proteins, the contamination may be caused by the ingestion of contaminated products. Further investigation regarding the sources of contamination and daily food processing and handling is needed to elaborate more on the relationship between those biological contaminants and stunting. Whether the contamination is site-specific is also interesting to be explored comprehensively.

*Prevotella*, *Butyrivibrio*, and *Muribaculaceae* were predominantly found in normal children. This microbiota was associated with low fat, high-fiber diet, carbohydrate intake, and increased propionate production. The *Alloprevotella* genus has been known for its anti-inflammatory effect on the gut and ability to produce butyrate [58].

Beta diversity metrics, Weighted and Unweighted UniFrac, demonstrated that nutritional status significantly influenced the microbiota diversity between observed groups. The present study showed a lower species richness (Chao1 alpha diversity metric) in stunted children than in normal children. This finding was also revealed in another study in India [59]. However, a dissimilar result from previous studies showed higher alpha diversity in stunted children [47] and no difference between stunted and normal children [60, 61]. Since it represents strong functional and ecological stability, higher alpha diversity is mainly found in a healthy or disease-improvement state [29]. Low-diversity gut microbiota environment was more susceptible to enteric infections [62]. However, different results in alpha diversity were relevant to the dynamic features of gut microbiota and their contributing factors.

The present study revealed a number of depleted and enriched genera in stunted children. However, dissimilar results showed the depleted abundance of *Blautia* and *Collinsella* and enriched abundance of *Akkermansia* [61], *Butyrivibrio* and *Alloprevotella* [47] in stunted children. Both studies revealed a higher abundance of *Copprococcus*. Meanwhile, the present study did not show a significant difference in the abundance of *Coprococcus* between observed groups. The exploration of the significant taxa based on their function revealed the beneficial and harmful effects of depleted and enriched genera in stunted children. *Enterococcus*, a commensal microbiota that was depleted in stunted children, played an important role in maintaining colonic homeostasis and decreasing the severity of infectious diarrhoea in children. It has been developed as a probiotic, although there were also some reports regarding hospital-associated infections [63]. *Terrisporobacter* and *Turicibacter* were enriched genera in healthy persons with normal glycemic levels [64]. *Asteroleplasma*, an enriched genus in stunted children, was also enriched in Type 2 Diabetes Mellitus and positively correlated to the increased HbA1c level [65]. *Lactobacillus*, a well-known probiotic genus, has been associated with intestinal inflammation, such as Crohn’s disease and Rheumatoid Arthritis. Whether *Lactobacillus* was directly involved in pathogenesis or adapted to survive the pro-inflammatory gut environment was poorly understood [66]. The present study showed an enriched abundance of *Anaerostipes* in stunted children. A similar result was also revealed in Pakistani stunted children, and *Anaerostipes* was negatively correlated to height [66]. However, other studies revealed the beneficial roles of *Anaerostipes*, especially regarding its ability to produce butyrate from the degradation of carbohydrates and commonly depleted in multiple diseases [67, 68]. Gut microbiota composition is dynamic and fluctuating in response to external factors and internal biological processes. Defining the ‘abnormal’ microbiome is challenging due to the temporal dynamics of gut microbiota [69], as well as distinct variations of gender, age, geographical, social-demographic, and medical contexts. Therefore, it was indispensable to investigate the ‘healthy’ microbiome in a proper setting [70].

This study discovered a higher expression of enteric pathogen virulence genes in stunted children than in normal children. To our knowledge, the virulence genes expression study in stunted children has never been reported. In the present study, the virulence genes not only existed but were also assuredly expressed, which later were translated into proteins and played critical roles in facilitating enteric infections. Gut inflammation contributed to the loss of microbial density and diversity, therefore supporting the growth of the Enterobacteriaceae family due to the lack of competition with other endogenous microbiota. The accumulation of Sulphur-containing by-product tetrathionate and nitrate from gut inflammation supported the rapid outgrowth of *Salmonella typhimurium* and *E. coli* [71]. The depletion of *Enterococcus* and *Alloprevotella*, which played essential roles in maintaining gut homeostasis and exhibiting anti-inflammatory effects, might promote susceptibility to enteric infections. However, this should be further explored.

Previous studies showed that the presence of EAEC harbouring *aaiC* gene was negatively correlated to linear growth in Bangladesh and Peru [72, 73]. It was explained that the correlations were site-specific [73]. EAEC and ETEC were the most prevalent causes of diarrhoea in children less than five years of age in China. Hot and warm temperatures contributed to the high prevalence [74].

The present study demonstrated a higher expression of ETEC’s heat-stabile toxin-encoded gene (*estA*) in stunted children than in normal children. ETEC was one of four diarrhoeal pathogens associated with moderate to severe diarrhoea cases among children under five in Asia and Africa. ETEC-associated stunting was reported in low-income and lower-middle-income countries, and it increased mortality [75]. A previous study showed that asymptomatic heat-stable toxin ETEC infections were significantly associated with childhood stunting [76].

This study only used *eaeA* to confirm the existence of EPEC among samples; therefore, it could not be differentiated whether the infections were typical or atypical. EPEC has been known as the most prevalent *E. coli* pathotype in children under five with diarrhoea [76]. A study in Bangladesh indicated that EPEC was associated with stunting and underweight [10].

*Shigella*/EIEC was the predominant cause of linear growth impairment in children with diarrhoea [77]. *Shigella* infections showed the strongest negative association with linear growth in children from developing countries [6] and increased mortality rate in stunted children [75].

This study used the *ompC* gene to reveal the existence of *Salmonella enterica* (*S. enterica*) among fecal specimens; however, the exact serovar was undetermined. A study showed that *Salmonella* was detected in fecal samples of stunted children in India using the culture method, although it was not statistically significant [78]. Another study revealed that non-typhoidal *Salmonella* infections were significantly associated with wasting but not stunting [11].

The present study highlighted the negative correlation between the expression of virulence genes, IGF-1 level, and height. IGF-1 through GH/IGF-1 axis is essential in stimulating the proliferation and differentiation of chondrocytes in the epiphyseal plate. IGF-1 also induces osteoblast proliferation, collagen secretion, and bone matrix mineralization, thus supporting linear growth [20–22]. The modulation by gut microbiota might take part in IGF-1 secretion, as previously reported in animal studies [17–19]. A previous study reported the correlation between diarrhoea due to enteric infections, IGF-1 concentration, systemic inflammation biomarker, and linear growth impairment in stunted children aged 6 to 24 months from Brazil [13].

Exposure to enteric pathogens caused EED. The changes in intestinal structures and functions and systemic inflammation have led to impairment of nutrient adsorption, growth hormone resistance, and disruption of bone growth and remodelling, thus resulting in linear growth faltering [32]. Diarrhoea magnified these conditions, especially by promoting nutrition depletion. Previous studies revealed the association between enteric infections and inflammation. ETEC, *Shigella*/EIEC, and EAEC were associated with increased EED scores in stunted children [73]. Previous studies revealed the association between enteric infections and the increase of inflammation markers, as follows: EAEC, EPEC, and ETEC were positively associated with the level of fecal calprotectin [10, 75]; *Shigella*, EPEC, and ETEC were positively associated with the levels of myeloperoxidase, and EIEC was positively associated with the levels of lactoferrin, interleukin (IL) 8, and IL-1b [79]. Acute *Salmonella* infection increased intestinal permeability. Chronic infection elevated the level of myeloperoxidase and activated the pro-inflammatory pathways, thus increasing the susceptibility to intestinal inflammation [80]. However, this study was unable to determine whether subjects exhibited the EED since the EED parameters were not evaluated.

Coinfection of enteric pathogens has been reported in pediatric diarrhoeal cases [81], and it might increase virulence. The existence of certain pathogens might support or facilitate others. A previous study showed that EAEC was coinfected with ETEC and EPEC [10]. The concurrence infections of enteric pathogens in children under five with diarrhoea predominantly happened in under 2-year-old children compared to 2–5 years of age [82]. The present study showed the existence of multiple enteric pathogen virulence genes. Whether the infections occurred in the same or different periods was undeterminable in this study.

The present study revealed a significant association between a history of diarrhoea and stunting. Furthermore, the expression of enteric pathogen virulence genes was higher in stunted children than in normal children. However, the lack of detailed information regarding types, onset, duration, diagnosis, and treatments of diarrhoea, as well as other clinical symptoms, was one of the limitations of this study. Recall bias from parents/guardians, as well as undocumented event or incomplete information regarding history of diarrhoea in Maternal and Child Report, were the major causes of the shortcomings. There was also no comprehensive survey regarding dietary intake (nutritional survey), which restricted the exploration of the association between dietary pattern and gut microbiota composition in stunted and normal children. An extensive study involving more subjects will provide more conclusive results regarding the gut microbiome profile in stunted and normal children and their role in linear growth.

## Conclusion

The present study showed that a history of diarrhoea and incomplete basic immunization status were strongly associated with stunting. It also discovered the distinctive gut microbiome composition in stunted and normal children from Pidie, Aceh, Indonesia. Gut microbiome profile revealed dysbiosis, a higher abundance of inflammation and metabolic abnormalities-related taxa, and unhealthy diets (high-fat/low-fiber diet). This study highlighted a higher abundance of water biological contaminants in gut microbiome of stunted children. The virulence genes expression study indicated a more vulnerable gut environment to enteric pathogens in stunted children, which might lead to linear growth retardation. These findings corroborate the importance of restoring dysbiosis and preserving the balanced state of gut microbiota in supporting linear growth in children. The existing stunting eradication programs need to be emphasized on several critical points. Prevention and adequate treatments of childhood diarrhoea and improvement of immunization coverage will provide better protection. Proper food and drinking water processing and adequate hygiene and sanitation are essential in reducing exposure to enteric pathogens. It must be persuasively and consistently educated to mothers and other food providers. Consuming healthy fermented food, adequate dietary fibers, and a proportional intake of fats, carbohydrates, and proteins may be beneficial in altering and shaping the balance of gut microbiota composition. Future research is encouraged to elaborate more on the relationship between water biological contaminants and linear growth impairment and explore the interactions/co-occurrences between enteric pathogens and other microbiota in stunted and normal children.

## Methods

### Study design and subject recruitment

This study was a case–control study involving 42 participants recruited using a purposive sampling method in Pidie District, one of the stunting pocket areas in Aceh Province, Indonesia (5°18′16.5"N 96°00′35.3" E). The sample size estimation was determined using the Lameshow formula for case–control study [83]. It was calculated based on the proportion of stunting in children under 5 years in Aceh Province, Indonesia (21.1%), as reported in Indonesia Basic Health Research in 2019 [84]. The protocol of this study was approved by the Research Ethics Committee of the Faculty of Medicine, Universitas Syiah Kuala (dossier No.050/EA/FK-RSUDZA/2021). Written informed consent was acquired from the parents or guardians of the participants in the presence of a third person. Study participants for the case (n = 21) and control groups (n = 21) were recruited based on the following criteria: weaned toddlers, 24–59 months of age, and no history of antibiotic treatment within the previous four weeks. Participants were of any gender or ethnicity and had no acute or chronic illnesses, symptoms, or congenital diseases. The length/height for age and weight for age for the case group was between -3 standard deviation (SD) and -2 SD of the median value of WHO Child Growth Standards. On the other hand, the value of length/height for age and weight for age for the control group was above -2 SD and below + 2 SD of the median value of standards, which meant that the participants were within the normal range and other types of malnutrition were excluded. The selected normal children for the control group were of the same age, sex, and living areas as the stunted children, with a 1:1 comparison. The anthropometric measurements of all participants were conducted in Puskesmas (community health center) facility from January to May 2021. The measurement was performed by trained health workers using standardized methods and equipment and extrapolated to stunting versus normal based on the WHO child growth standard [28]. Kartu Menuju Sehat (Health Card) and Buku Kesehatan Ibu dan Anak (Maternal and Child Report) was used to confirm the health status of the subjects, including the record of growth, health and immunization status. History of diarrhoea was collected using direct interview with parents/guardians. It referred to previous recurrent incidents of acute diarrhoea with three or more bloody or watery stools daily and happened for < 7 days with or without hospitalization. Basic immunization status was indicated as the fulfilment of mandatory vaccinations (Expanded Programs of Immunization/EPI) of participants from 0 to 23 months consisted of Bacille Calmette-Guérin (BCG), Hepatitis B, diphtheria, tetanus, pertussis-*Haemophilus influenzae* type B-Hepatitis B (DTP-Hib-HepB), Polio, and Measles.

### IGF-1 measurement using ELISA

The sera isolated from the whole blood were stored at − 20 °C and used for the immunochemistry analysis to measure the level of human IGF-1 (Human IGF-1 ELISA Kit, Sigma-Aldrich) using the sandwich ELISA method. The intensity of the colour was measured by spectrophotometer (Multiskan Go, Thermo Scientific) at 450 nm. The amounts of IGF-1 were derived by interpolation from the reference/standard curve generated from the known concentrations of IGF-1 in the same assay. The results of unknown samples were calculated using the four-parameter logistic function [85].

### 16S rDNA amplicon sequencing

About 1 g of the faecal specimen for each sample was mixed into 9 ml DNA-RNA shield solution (Zymo Research, USA) and stored at -80 °C until further used for DNA and RNA extraction. The DNA from fecal specimens was extracted using Quick-DNA Fecal/Soil Microbe Miniprep Kit (Zymo Research). The genomic DNA for metagenomics analysis was ensured to meet several requirements such as amount (≥ 200 ng), concentration (≥ 20 ng/µl), volume (≥ 12 ul), and purity (OD 260/280 = 1.8–2.0, with no degradation or contamination). The amount of DNA was quantified using Nanodrop Spectrophotometer (Maestrogen). The degradation of DNA was assessed using agarose gel electrophoresis. The DNA extraction was performed by PT. Genetika Science Indonesia, a metagenomics services provider. The gut microbiota profiling was determined using NGS (Illumina NovaSeq 6000, Novogene), which targets the V3-V4 region of bacterial 16S rDNA. The V3-V4 region was amplified using forward primer 341F 5’-CCTAYGGGRBGCASCAG-3’ and reverse primer 806R 5’- GGACTACNNGGGTATCTAAT-3’ [86, 87]. The metagenomics analysis workflow consisted of amplification using Polymerase Chain Reaction (PCR), library preparation, sequencing, and Bioinformatics analysis. Quality control was performed in all steps to ensure the quality and validity of data. Bioinformatics analyses were conducted using the microbiome bioinformatics platform QIIME2 version 2022.11 (https://qiime2.org) in processing the reads, picking, and classifying the ASV (SILVA version 138) as well as measuring microbiome diversity. The prevalence cut-off was set to 10%, which meant that ASVs found in fewer than 10% of total samples were removed.

### Virulence genes expression analysis

The RNA from fecal specimens was extracted using RNAeasy Power Microbiome Kit (Qiagen). The process was optimized to fulfil the requirement, such as purity (OD 260/280 was 1.8–2.0 and A260/230 was above 2) and sufficient concentration for expression study. The RNA was used as a template for cDNA synthesis (ReverTraAce with gDNA Remover, Toyobo). Each reaction with 10 µl of total volume consisted of 150 ng RNA, 2 ul 4 × DN Master Mix (contained DNAse I), and 2 ul 5 × RT Master Mix II. The cDNA synthesis program consisted of denaturation of RNA at 65 °C for 5 min, followed by genomic DNA removal at 37 °C for 5 min, cDNA synthesis at 37 °C for 15 min, and final heat at 98 °C for 5 min.

The expression study was conducted by quantitative real-time PCR (CFX96 Touch Real-Time PCR, BioRad) using DNA-binding fluorescent dye/SYBR Green (SensiFAST SYBR® No-ROX, Bioline). The amplifications were carried out in triplicate. Each reaction (20 µl of total volume) consisted of 10 µl of 2 × SensiFAST SYBR® No-ROX Mix (Bioline), forward and reverse primers, 1 µl cDNA, and nuclease-free water. The amplification program consisted of polymerase activation at 95 °C for 2 min, followed by 40 cycles of denaturation at 95 °C for 5 s, annealing for 10 s, and elongation at 72 °C for 20 s. The bacterial 16S rDNA gene (*rrs*) was used as a reference gene and it has shown a stabile performance in observed groups (Additional file 1: Table S1). Since the efficiency values of qPCR reaction using the set of primers were not equal to 2, the relative quantification was calculated using the Pfaffl method with efficiency-corrected calculation [88]. The list of primers used in this study was shown in Additional file 1: Table S2.

### Statistical analyses

The difference between groups in anthropometric values and alpha diversity Shannon index were analysed using an independent T-test. The categorical data were analysed using Fisher Exact Test. The level of IGF-1 and Alpha diversity Chao1 were analysed using Mann–Whitney U Test due to the non-normal distribution of data. Beta diversity analysis was performed using PERMANOVA with Weighted and Unweighted UniFrac models. The differential abundance analysis of statistically significant ASV between groups (normal vs. stunted) was performed using Linear Discriminant Analysis Effect Size (LEfSe). The association of taxonomic abundance and nutritional status was calculated using the Mann–Whitney U Test and corrected using False Discovery Rate (FDR) by Benjamin-Hochberg with a significant threshold of 10%. The correlation between virulence gene expression and nutritional status was evaluated using Mann–Whitney U Test. The correlation matrix between virulence genes expression, IGF-1 sera level, and height was constructed using the Spearman-rank test. All statistical analyses were performed using the R software package version 4.3.0 [89]. The results of statistical analyses were visualized using R studio version 2023.03.0 [90] and GraphPad Prism version 8.4.3.

### Supplementary Information

**Additional file 1: Fig. S1.** Unweighted UniFrac model for beta diversity. The metric was visualized using QIIME2 emperor. **Fig. S2**. Rarefaction curve of alpha diversity. The minimum sample depth was used as rarefaction depth (51,636 reads). The curve were plotted using Ampvis2 package in R and visualized in RStudio. **Table S1**. The reference gene (*rrs*) expression among observed groups. **Table S2**. Primers used in this study.

**Additional file 2**. Taxonomy table of classified taxa from 16S rDNA amplicon sequencing. The table was constructed and converted using QIIME2. Enteric infections-related taxa were bolded.

## Data Availability

The datasets supporting the conclusions of this article are included within the article and its additional files. Further request for information can be directed to the corresponding author.

## Abbreviations

IGF-1: Insulin-like Growth Factor 1
ELISA: Enzyme Linked Immunosorbent Assay
rDNA: Ribosomal Deoxyribonucleic acid
qPCR: Quantitative Polymerase Chain Reaction
EAEC: Enteroaggregative *Escherichia coli*
ETEC: Enterotoxigenic *Escherichia coli*
EPEC: Enteropathogenic *Escherichia coli*
EIEC: Enteroinvasive *Escherichia coli*
EED: Environmental Enteric Dysfunction
BMI: Body Mass Index
BCG: Bacille Calmette-Guérin
DTP-Hib-HepB: Diphtheria, tetanus, pertussis-*Haemophilus** influenzae* type B-Hepatitis B
LEfSe: Linear discriminant analysis effect size
LDA: Linear Discriminant Analysis
GH: Growth hormone
CYN: Cylindrospermopsin
SD: Standard seviation
WHO: World Health Organization
ASV: Amplicon Sequence Variant
OD: Optical density
FDR: False discovery rate
