# Supplementary material for: Correlation between gut microbiota composition, enteric infections and linear growth impairment: a case–control study in childhood stunting in Pidie, Aceh, Indonesia
Source: Gut Pathog. 2023 Nov 9;15:54. doi: 10.1186/s13099-023-00581-w (PMC10636988; doi:10.1186/s13099-023-00581-w)
Supplement: Supplementary file 1 — Additional file 1: Fig. S1. Unweighted UniFrac model for beta diversity. The metric was visualized using QIIME2 emperor. Fig. S2. Rarefaction curve of alpha diversity. The minimum sample depth was used as rarefaction depth (51,636 reads). The curve were plotted using Ampvis2 package in R and visualized in RStudio. Table S1. The reference gene (rrs) expression among observed groups. Table S2. Primers used in this study. [file 13099_2023_581_MOESM1_ESM.docx]

**Additional file 1**


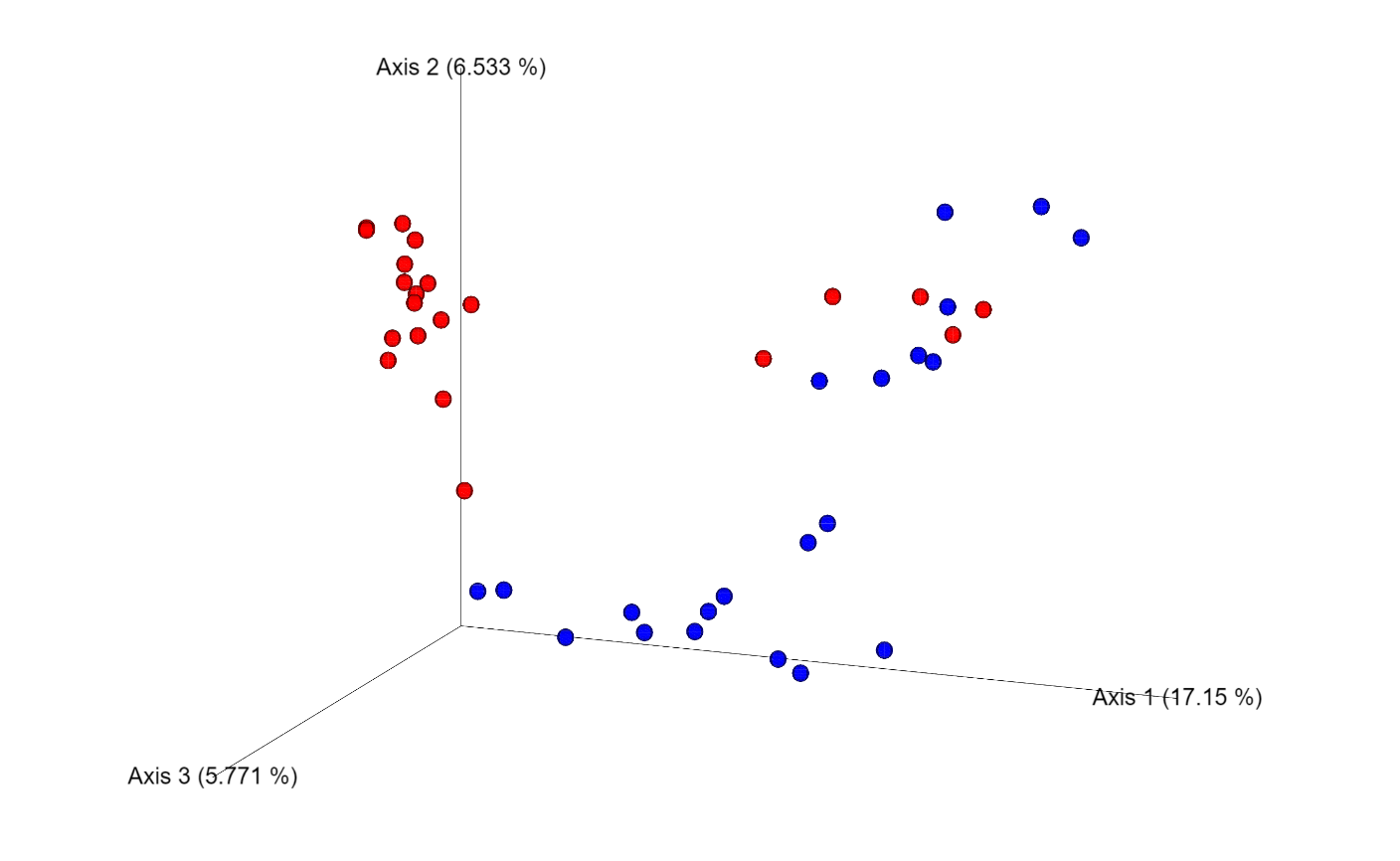

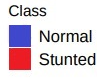


**Fig. S1** Unweighted UniFrac model for beta diversity. The metric was visualized using QIIME2 emperor.


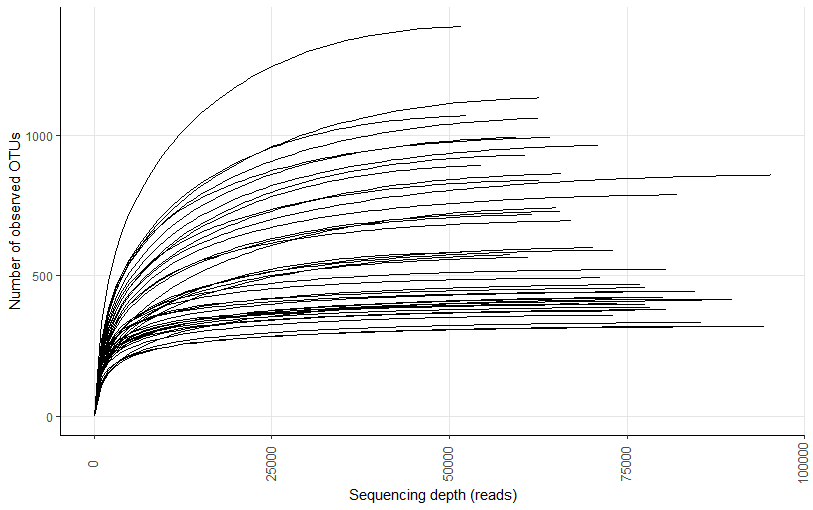


**Fig. S2** A rarefaction curve of alpha diversity. The minimum sample depth was used as rarefaction depth (51,636). The curve were plotted and visualized using QIIME2.

**Table S1. The reference gene (*rrs*) expression among observed groups**

| Parameter | Observed groups | |
| --- | --- | --- |
|  | Normal | Stunted |
| Mean Cq* | 15.15 | 15.95 |
| Standard Deviation | 0.20 | 0.19 |
| %CV | 1.35 | 1.22 |
| ***amplification was conducted in triplicate** | | |

**Table S2. Primers used in this study**

| **Targeted genes/Enteric pathogens** | **Primers (5’-3’)** | **Product size** | **Primer concentration**  **(nM)** | **Ta**  **(°C)** | **Efficiency (%)** | **References** |
| --- | --- | --- | --- | --- | --- | --- |
| *aaiC/*Enteroaggregative *Escherichia coli* (EAEC) | aaiC-F: ATTGTCCTCAGGCATTTCAC | 215 | 400 | 54 | 98 | [1] |
|  | aaiCR: ACGACACCCCTGATAAACAA |  |  |  |  |  |
| *eaeA/*Enteropathogenic  *Escherichia coli* (EPEC) | eaeA-F: GGTCAGATTCAGCATAGCGG | 103 | 500 | 58 | 102 | [2] |
|  | eaeA-R: CGCGAGCGGTCACTTTATAA |  |  |  |  |  |
| *estA/*Enterotoxigenic *Escherichia coli* (ETEC) | STh-F: TTCACCTTTCGCTCAGGATG | 166 | 500 | 58 | 101 | [2] |
|  | STh-R: CCCGGTACAAGCAGGATTAC |  |  |  |  |  |
| *IpaH-3/Shigella* and Enteroinvasive *Escherichia coli* (EIEC) | IpaH3-F: ACAGGTGATGCGTGAGACTG | 101 | 400 | 60 | 100 | [3] |
|  | IpaH3-R: ATGAATGGTGCAGTYGTGAG |  |  |  |  |  |
| *OmpC/Salmonella enterica* | ompC-F: ACCGCTAACGCTCGCCTGTAT | 122 | 400 | 54 | 107 | [1] |
|  | ompC-R: CGGGTTGCGTTATAGGTCTGA |  |  |  |  |  |
| *rrs/ Bacterial* 16S rDNA  (reference gene) | Tbac-F: GTGSTGCAYGGYTGTCGTCA | 110 | 400 | 65 | 100 | [4] |
|  | Tbac-R: ACGTCRTCCMCACCTTCCTC |  |  |  |  |  |
| **Degenerated nucleotide: S = G or C, Y = C or T, R = A or G, M = A or C** | | | | | | |

References:

1. Liu J, Gratz J, Amour C, Nshama R, Walongo T, Maro A, et al. Optimization of quantitative PCR methods for enteropathogen detection. PLoS One. 2016;11:1–11.
2. Flaherty KE, Grembi JA, Ramachandran V V., Haque F, Khatun S, Rahman M, et al. High-throughput low-cost nl-qPCR for enteropathogen detection: A proof-of-concept among hospitalized patients in Bangladesh. PLoS One. 2021;16:1–13.
3. Barbau-Piednoir E, Denayer S, Botteldoorn N, Dierick K, De Keersmaecker SCJ, Roosens NH. Detection and discrimination of five E. coli pathotypes using a combinatory SYBR® Green qPCR screening system. Appl Microbiol Biotechnol. 2018;102:3267–85.
4. Casañas MA, Rangkasenee N, Krattenmacher N, Thaller G, Metges CC, Kuhla B. Methyl-coenzyme M reductase A as an indicator to estimate methane production from dairy cows. J Dairy Sci. 2015;98:4074–83.
